# Investigations concerning the application of the cross-correlation method in cardiac output measurements

**DOI:** 10.1186/1475-925X-11-24

**Published:** 2012-05-20

**Authors:** Maciej Gawlikowski, Tadeusz Pustelny

**Affiliations:** 1 Foundation of Cardiac Surgery Development, Artificial Heart Lab., Zabrze, Poland; 2 Silesian Technical University, Faculty of Electrical Engineering at the Silesian University of Technology, Gliwice, Poland

**Keywords:** Heart diagnostic, Cardiac output measurement, Thermodilution method, The cross-correlation method

## Abstract

**Background:**

In spite of numerous non-invasive examinations the “gold clinical standard” of cardiac output measurements is the invasive pulmonary artery catheterization by means of the Swan-Ganz catheter and the application of the thermodilution method to estimate the blood flow. The results obtained by means of thermodilution are sensitive to many physical and biological disturbances. The unreliability of this method amounts to 20-45% and depends on the given variant of the method. Therefore some other method, more accurate and resistant to disturbances, was looked for. This paper presents a new approach to cardiac output measurements, based on cross-correlation signal analysis. The goal of investigations was to verify experimentally the application of the cross-correlation method of cardiac output measurements.

**Results:**

In 99.2% of the examined cases the extreme of the cross-correlation function was easy to be estimated by numerical algorithms. In 0,8% of the remaining cases (with a plateau region adjacent to the maximum point) numerical detection of the extreme was inaccurate. The typical unreliability of the investigated method amounted o 5.1% (9.8% in the worst case). Investigations performed on a physical model revealed that the unreliability of cardiac output measurements by means of the cross-correlation method is 3–5 times better than in the case of thermodilution.

**Conclusions:**

The performed investigations and theoretical analysis have shown, that the cross-correlation method may be applied in cardiac output measurements. This kind of measurements seems to be more accurate and disturbance-resistant than clinically applied thermodilution.

## Background

The most fundamental hemodynamic parameter is the cardiac output (*CO*). It is defined as the average blood flow which is pumped through the cardiac muscle in the course of one minute [1,2]. For over 40 years pulmonary artery catheterization (*PAC*) and *CO* measurements by means of thermodilution constituted the “gold standard” of hemodynamics diagnostics [2-6]. Nowadays, invasive examinations are slowly taken off the intensive care wards and the no-invasive one (e.g. echocardiography) or minimally invasive (e.g. PiCCO [7], LidCO [8]) have been put into practice. It should be emphasized that non-invasive methods of *CO* measurements are recognized as less accurate [2,3,6,8] and minimally invasive methods must be calibrated before examination by means of thermodilution [7,8]. Actually there are a few methods based on other physical principles [9,10], but nowadays they are being developed. The *PAC* technique by means of the Swan-Ganz catheter allows to obtain or calculate other valuable hemodynamic parameters basing on intracardiac pressures, e.g. pulmonary artery wedge pressure, pulmonary resistance etc. [2,6]. It has been evidently proved that invasive *PAC* examinations did not increase the mortality rate [11]. Therefore, in spite of their invasiveness, *PAC* and *CO* measurements by thermodilution still constitutes the “gold standard” and they are frequently utilized in intensive care wards.

The *PAC* method requires the insertion of a special catheter (Swan-Ganz catheter) into the pulmonary artery [1-3,6]. A miniaturized thermistor is located at the tip of the catheter. The examination runs as follows: first the indicator (mostly iced or room-temperature 0.9%NaCl) is injected into the right atrium by a duct located in the interior of the catheter. Next, the temperature variations triggered by the indicator are measured by the thermistor and the indicator dilution curve (*IDC*) is registered by an external computer (most often by a patient monitor). The *CO* value is calculated by means of the Stewart-Hamilton formula **(1)**. The estimation of the unreliability of the thermodilution method is rather complicated because of the lack of other reference methods or means of significant influences on the examined object (human or experimental animal) [8,12-14]. Therefore the accuracy is determined theoretically. Comprehensive investigations of the unreliability of the thermodilution method (theoretical and performed on a physical model) revealed a high susceptibility of this method to disturbances and a low repeatability of measurements. The typical unreliability of “iced” and “room-temperature” thermodilution proved to amount to 18% and 27%, respectively (the worst cases were 34% and 54%, respectively) [13,15].

(1) $$Q=\frac{c_{i}\cdot \sigma_{i}}{c_{b}\cdot \sigma_{b}}\cdot \frac{V_{i}\cdot \left(T_{b}-T_{i}\right)}{\int_{0}^{T}T_{b}(t)dt}$$

where: *c*_*b*_*, c*_*i*_ – specific heat of the blood and indicator, *ρ*_*b*_*, ρ*_*i*_ – mass density of the blood and indicator, *V*_*i*_ – indicator volume, *T*_*b*_*, T*_*i*_ – temperature of the blood and indicator.

### Goal

In spite of certain imperfections, thermodilution is essentially a clinical method of *CO* measurements. Low accuracy and repeatability cause that prospecting other measurement methods, based on other physical principles, is justified.

One of the flow measurement methods (occasionally applied for atypical liquid or intricate objects) is the cross-correlation [13,16,17]. The goal of investigations was to verify experimentally the application of the cross-correlation method for cardiac output measurements.

## Methods

### Background of cross-correlation flow measurements

**Figure 1:**
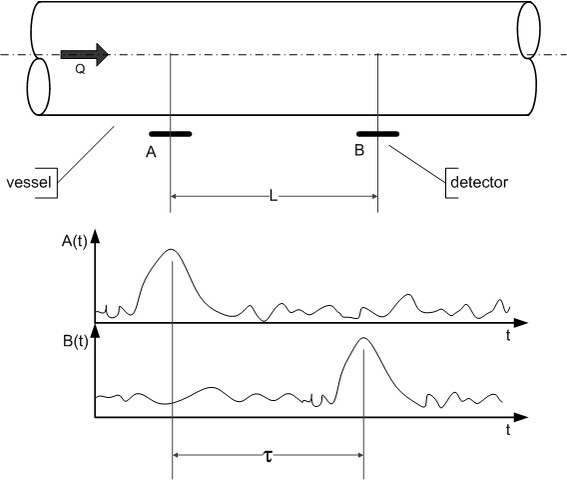
The idea of cross-correlation flow measurements.

Let us consider the section of a vessel in which the liquid flows with an average velocity *v*_*AV*_ (see Figure 1). A disturbance (naturally or artificially inducted) of the flow morphology appears at point *A* and moves to point *B* without any change of its form. The disturbance is detected at the points *A* and *B*. The distance between the sensors amounts to *L*. Supposing, that the stochastic process is ergodic and stationary and that the disturbance does not change its form between the points *A* and *B*, the signal registered in point B may be defined by **(**2**)**:

(2) B(L,t+τ)=A(0,t)

where: *τ* – transit time of disturbance.

The cross correlation function *R*_*AB*_ calculated at *A(t)* and *B(t)* signals proves to achieve its extreme at *t* = *τ*[17]:

(3) $$\begin{array}{l} R_{\mathrm{AB}}(\tau )=\frac{1}{T}\int_{0}^{T}A(0,t)\cdot B(L,t+\tau )dt\\ \tau \equiv \max[R_{\mathrm{AB}}(\tau )] \end{array}$$

where: *T* – finite time of integration.

The velocity of the disturbance can be calculated from **(**4**)**:

(4) $$v=\frac{L}{\tau }$$

The relationship between the transit time of disturbance and its velocity is more complex and depends on the spatial distribution of the flow. At a laminar flow through the cylindrical vessel the spatial distribution of the flow velocity is parabolic [18]. For this kind of flow the average velocity *v*_*AV*_ equals half the maximum velocity *v*_*max*_.

**Figure 2:**
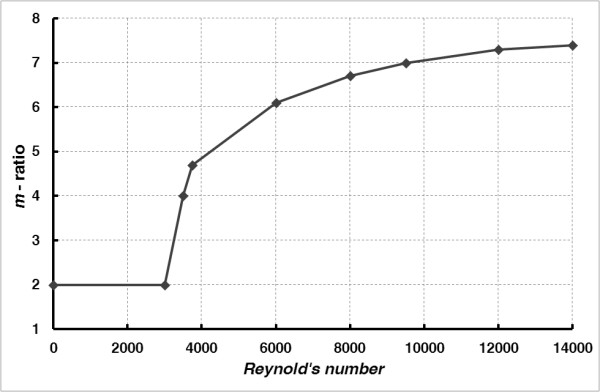
***m-*ratio vs. Reynold’s number dependence.**

In the case of a turbulent flow the velocity distribution can be approximated, for example, by means of Prandt’s formula [18] or by applying the more adequate equation **(**5**)**[19]. The value of the parameter *m* depends on the character of the flow defined by Reynold’s number and is presented in Figure 2.

(5) $$\begin{array}{ll} v(x) & =v_{\max}\left[1-{\left(\frac{x}{R}\right)}^{m}\right]\\ & =v_{\mathrm{AV}}\cdot \frac{m+2}{m}\cdot \left[1-{\left(\frac{x}{R}\right)}^{m}\right] \end{array}$$

where: *x* – distance from the centre line of the vessel, *R* – radius of the vessel, *v*_*max*_ – flow velocity at the centre line of the vessel.

The definition of the average velocity is of fundamental importance for the estimation of the volumetric flow, e.g. in cardiac output measurements. The average flow velocity is defined as the velocity at which the volumetric flow of the liquid with a certain spatial distribution equals the volumetric flow of the liquid with a uniform spatial distribution [18]. The knowledge of the value of the mean velocity allows to calculate the volumetric flow through a vessel with a known diameter, by means of **(**6**)**.

(6) $$Q=v_{\mathrm{AV}}\cdot A$$

where: *Q* – volumetric flow, *v*_*AV*_ – average flow velocity, *A* – area of the cross section of the vessel.

### The procedure of the experiment

**Figure 3:**
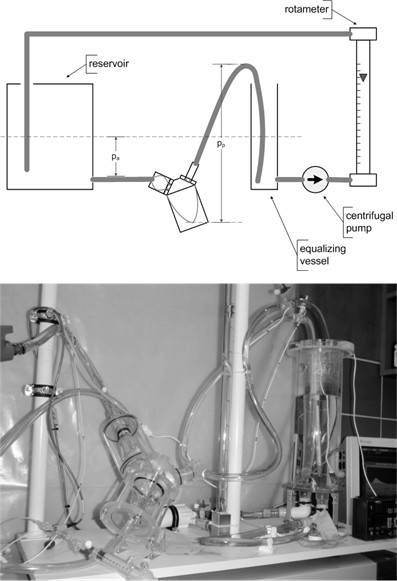
The physical model of pulmonary circulation.

**Figure 4:**
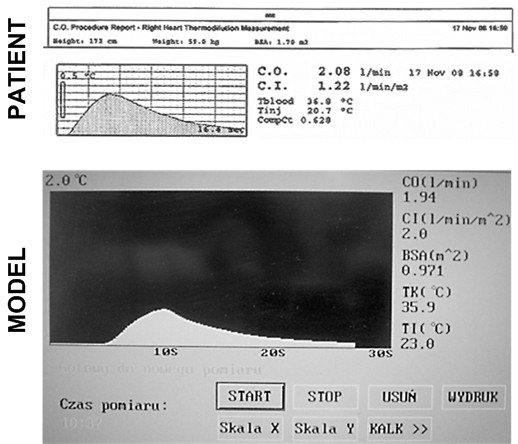
The comparison of IDC obtained from the patient and the model (for blood flow 2.0 L/min).

All measurements were performed on a physical model, which could simulate the essential phenomena of thermodynamic and fluid mechanics [13,14,20]. The schematic diagram and the pictorial view of the model have been presented in Figure 3. The high fidelity and usefulness of this apparatus to simulate the dilution of the thermal indicator was proved by comparing the *IDC* obtained (at the same flow rates and examination conditions) of the patient and the model (Figure 4).

Experiments were performed on the pulsating flow at the following conditions: heart rate 40–70 beats per minute, duty cycle 25-50%.

**Figure 5:**
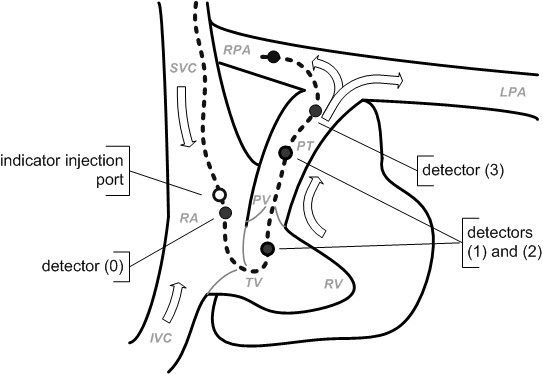
Measurement points of disturbance movement.

During the experiments the thermal disturbances were generated artificially by injecting 3, 5 or 10 mL of the indicator (0.9%NaCl, temperature 2-7°C or 21-26°C). Those volumes of the indicators are typical for the thermodilution method (3 and 5 mL for infants and 10 mL for adults [6]). It is known from the theory of ergodic and stationary stochastic processes, that the shape of disturbance itself does not influence the cross-correlation function (on condition, that the form of the disturbance was not distorted between the detectors) [16]. Therefore, the way of injecting was not measured. As detectors four clinical Swan-Ganz catheters (Becton-Dickinson) were used. The catheters were modified by cutting the distal part close to the thermistor. The detectors were located in the physical model of pulmonary circulation at the following points: right atrium *(0)*, right ventricle *(1)*, pulmonary artery *(2)* and at the pulmonary artery bifurcation *(3)* (Figure 5). The distance between the detectors *(0)* and *(3)* was 35 cm. The disturbance was excited in the right atrium. As the operation fluid 30% water-glycerine solution was applied (mass density *ρ* = 1067 kg/m^3^, absolute viscosity *μ* = 0.004 Pa·s). During the experiments the temperature of the operation liquid was stabilized in the range of 36.5 – 37.1°C.

After the injection of the indicator the signals originated by the sensors *(0)*…*(3)* were registered (sampling frequency 1 kHz). Next, the registered signals were processed (LabVIEW, SignalProcessing Toolkit) in the following way: filtering (digital Bessel low-pass filter, cutoff frequency 0.25 Hz), normalization, cross-correlation calculation (Fourier’s method), searching for the cross-correlation function extreme, estimation of the transit time *τ* of the disturbance. The extreme of the cross-correlation function was determined by the differential method. The last step was the calculation of the volumetric flow value by means of the algorithm described below:

● step 1: calculation of *v*_*max*_ value from **(4)** – signals from the detectors *(0)* and *(3)* were analyzed,

● step 2: calculation of Reynold’s number – the maximum value of spatial profile of flow velocity *v*_*PTmax*_ was calculated by means of **(**4**)**. The transit time *τ*_*23*_ was calculated as the difference between *τ*_*03*_ and *τ*_*02*_. Those signals are received by detectors located in a vessel with a constant diameter, as opposed to the sensors *(0)* and *(1)*, which are located in the atrium and ventricle. For calculations of the value *Re* the following medium parameters were assumed [13,20]: mass density *ρ* = 1067 kg/m^3^, absolute viscosity *μ* = 0.004 Pa·s. The *v*_*PTmax*_ is a maximum value of the continuous flow velocity. Because of the pulsatory character of the flow, the actual value of *v*_*PTmax*_ is greater than for the continuous one and it depends on the duty cycle. Therefore *v*_*PTmax*_ was corrected by the factor *k*_*p*_ and the respective equations are presented below:

(7) $$v_{PT\max}=k_{p}\cdot \frac{L_{23}}{\tau_{23}}$$

where: *k*_*p*_ – correction factor, *L*_*23*_ – distance between the detectors (2) and (3), *τ*_*23*_ –transit time of the disturbance between the detectors (2) and (3).

(8) $$k_{p}=\frac{T_{\mathrm{SYS}}}{T}$$

where: *T*_*SYS*_ –ejection time of the liquid during its pulsatory flow, *T* – period of flow pulsation.

● step 3: from *Re* the value of the factor *m* in **(**5**)** is estimated, basing on Figure 2,

● step 4: the average flow velocity *v*_*AV*_ is calculated making use of the equation **(**5**)**,

● step 5: the volumetric flow is calculated by means of **(**6**)**.

## Results

In the applied physical model of pulmonary circulation the pulmonary trunk diameter was *D*_*PT*_ = 1.3 cm. As the liquid flows through elements with different diameters (right ventricle and pulmonary trunk), the equivalent diameter of the vessel was assumed to be *A*_*z*_ = 4 cm^2^[13].

The total amount of the measured flow set points was *N* = 31. For all set points *n* = 8 the transit time was measured. The measurements were performed for a flow in the range of 1.3 – 4.0 L/min (the corresponding average and maximum flow velocity was from 0.10 to 0.26 m/s and 0.20 – 0.55 m/s, respectively). Those values correspond to the maximum flow velocity through the pulmonary artery valve (0.6 m/s, refer to [1]). At these conditions the Reynolds number was in the range of 700 – 2200.

**Figure 6:**
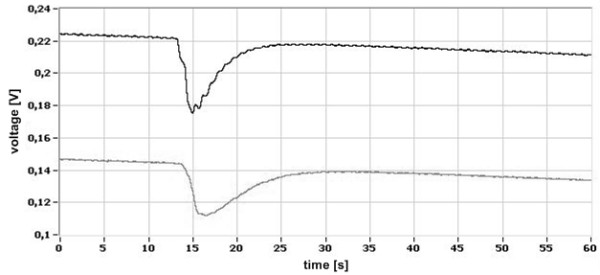
**Raw signals of disturbance: the upper one - from the detector *(0)*, the lower one - from detector *(3)*.**

**Figure 7:**
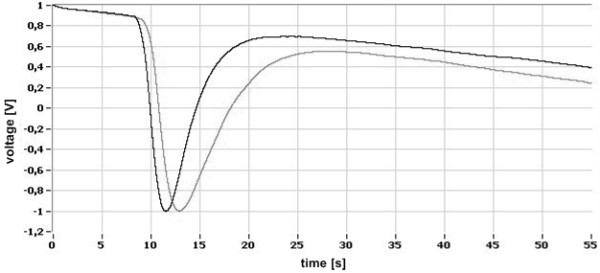
Filtered and normalized signals of disturbance.

**Figure 8:**
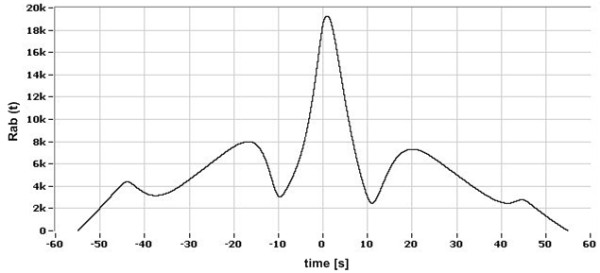
Cross-correlation function.

The demonstration signals received by the sensors *(0)* and *(3)* in the consecutive stages of signal processing are presented in Figures 6, 7, 8.

In 99.2% of the examined cases the extreme of the cross-correlation function was easy to be estimated by numerical algorithms. In 0,8% of the remaining cases (with a plateau region adjacent to the maximum point) numerical detection of the extreme was inaccurate.

**Figure 9:**
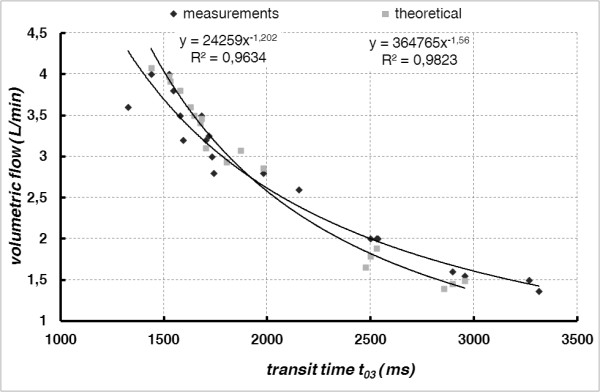
Comparison of theoretical and experimental transfer functions of cross-correlation methods.

In Figure 9 the measured and theoretically calculated transfer function (volumetric flow *vs.* measured disturbance transit time) of the investigated cross-correlation method is being compared.

According to [21] there is no unequivocal definition of the uncertainty of measurements and the notion of unreliability must be clearly defined with reference to the specific process of measurements. For the presented results it was defined as a relative value of deviation between transit times: measured and calculated by means of approximation to the power function [21]. The results of calculations of the unreliability of the cross-correlation method have been gathered in Table 1.

**Table 1:** Unreliability of the cross-correlation method

**Unreliability**	**Value**	**Remarks**
Average	5.1%	Standard deviation = ±3.5%
The worst case	9.8%	For fuzzy maximum of the cross-correlation function

## Discussion

One of the assumptions of the cross-correlation method is the immutability of the disturbance signal form received by the detectors [16,17]. In the performed investigations the disturbance was excited artificially by injecting the indicator. In this case the shape of the disturbance signal is modified mainly by a diffusive dilution of the indicator. This phenomenon was discussed theoretically [13] and defined in the form of the stochastic *Local Diffusion Random Walk* model of dilution [13,15]. Actually, the effect of the modification of the shape of the disturbance signal can be observed on raw signals received by the detectors (see: Figure 6). In spite of that a sharp extreme is to be observed in the cross-correlation function (Figure 8). Only in 0.8% of the analyzed cases the maximum of the cross-correlation function was fuzzy, resulting in a poor accuracy of estimation of the transit time. It was proved [17] that the recognition of the function extreme by means of the weighted mean method allows to achieve a 30% lower systematic error than by simple max. or min. searching (e.g. by differentiation of the analysed function).

Transit times lower than 420 ms were immeasurable by means of the presented method. The reason of this effect has not been explained unambiguously (low sampling frequency and near-field of thermal disturbance were taken into account). Therefore, in order to estimate of *Re*, *τ*_*23*_ was calculated as a *τ*_*03*_ – *τ*_*02*_. The transit time *τ*_*03*_ was well-measurable and correlated with the reference flow. The *τ = f(Q)* dependence is non-linear but it may be precisely approximated by the power function (*R*^*2*^ = 0.961). The reason of nonlinearity has not been unambiguously explained and the best fitting by power function was found experimentally.

The obtained results were to a high extent independent of temperature and the volume of the injected indicator. It appeared (see Table 2), that in the same hydrodynamic conditions the typical unreliability of cardiac output measurements according to the cross-correlation method is 3–5 times lower than in the case of thermodilution [12-15]. It should be emphasized, that investigations were performed on the pulsating flow only (heart rate 40–70 bit per minute, duty cycle 25-50%). The results of researches confirmed earlier opinions about the low significance of the flow pulsation for measurements taken by means of the cross-correlation method [16]. This fact may be essential in case of possible applications of the presented method in cardiac output measurements.

**Table 2:** Unreliability of various cardiac output measurement methods comparison

**Method**	**Unreliability**	
	**Typical**	**The worst case**
Iced thermodilution	18.3%	26.9%
Room-temperature thermodilution	34.2%	54.7%
Cross-correlation method	5.1%	9.8%

**Figure 10:**
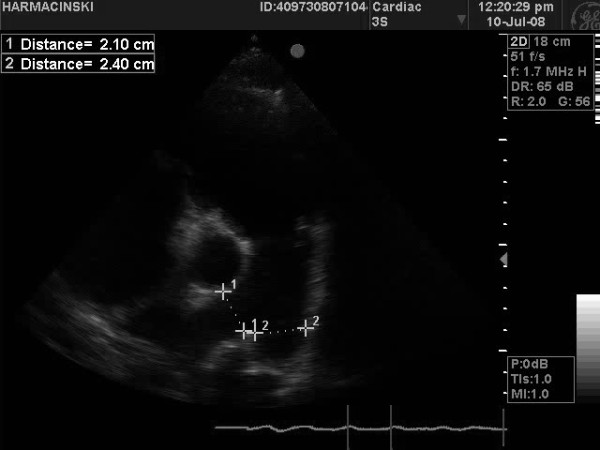
The demonstration of pulmonary artery trunk diameter measurement (tranthoracic echocardiography).

The advantages of the presented cross-correlation method of *CO* measurements in comparison with thermodilution are: a better accuracy and higher resistance to disturbances. The method may be carried out by a simple modification of the typical construction of the Swan-Ganz catheter (one extra thermistor must be added), which allows to retain the possibility of intracardiac pressure measurements. A disadvantage of *CO* measurements by means of cross-correlation is the need of estimating the pulmonary trunk (*PT*) diameter. This may be performed by transthoracic echocardiography (one of examinations performed by the authors has been presented in Figure 10) or by assuming the estimated diameter of this vessel basing on other factors (e.g. age, gender, *BMI*, diameter of other vessels – additional investigations are required).

There are many other methods of *CO* measurements and some of them have been applied clinically. Among non-invasive methods the most significant one is echocardiography. In Doppler’s echocardiography the *CO* is calculated by means of the blood velocity [13,22]. The spatial profile of the flow of blood through the specific vessel is unknown (in calculations a laminar flow is assumed), therefore the results of measurements are inaccurate. Another approach is based on the estimation of the left ventricle volume by means of its two-dimensional cross section [13,23]. The estimation is realized by rough equations which influents the uncertainty of measurements. Another non-invasive method is rheocardiography [3,5]. The method itself was designed for continuously monitoring of tthe hemodynamic parameters in healthy patients (aircraft pilots). It was clinically proved [5] that with reference to patients with cardiac a illness this method is suitable for monitoring long-term tendency rather than isolated measurement. Most of the modern minimally invasive methods based on the pulse contour analysis (e.g. PiCCO [7] and LidCO [8]) assume an immutable transfer function between excitation (blood flow generated by the heart) and response (pressure in peripheral artery). Generally this transfer function is unknown; therefore, its empirical estimation by means of other methods (most often by thermodiluion) is indispensable.

In fact, the accuracy of the methods mentioned above is unknown. In many papers only the correlation of the investigated method with the reference is estimated. Mostly, the reference constitutes thermodilution (or - hardly ever - the Fick method) with a typical accuracy in the range of 18-35% [2,12,13].

The application of the cross-correlation method is not confined to cardiac output measurements only. One of the most fundamental problems connected with the mechanical heart supporting therapy is the estimation of the flow generated by the heart prosthesis [9,24-27]. The cross-correlation method with a spontaneously excited disturbance (e.g. the blood pressure variation produced by artificial valves) may be applied in ventricular assisting devices.

## Conclusions

The performed investigations and theoretical analysis have shown, that the cross-correlation method may be applied in cardiac output measurements. This kind of measurements seems to be more accurate and disturbance-resistant than clinically applied thermodilution. The idea of cross-correlation cardiac output measurements has been patented [15] (patent pending).

The presented investigations were performed on one geometry of physical models of heart and vessels. Therefore the obtained results should be treated as preliminary ones. Investigations should be continued on other geometries and – in future – on animals.

## Competing interests

The authors declare that they have no competing interests.

## Authors’ contributions

MG developed the physical model of pulmonary circulation and performed investigations on thermodilution and cross-correlation. TP developed the cross-correlation flow measurement and analyzed the obtained data. Both authors have read and approved the final manuscript.
